# Assessment of the Safety, Efficacy, and Benefit of Empagliflozin in Patients With Type 2 Diabetes Mellitus (T2DM) and Heart Failure With Reduced Ejection Fraction (HFrEF) at High Risk for Cardiovascular Events

**DOI:** 10.7759/cureus.33070

**Published:** 2022-12-28

**Authors:** Ruba Towiargi, Lama Fetyani, Naila Aljahdali, Adnan Alnofeie, Yahya Alnoamy, Reham Ghandorah, Abrar Abduljawad, Njood Alharbi, Alanoud Alghanmi, Hala AlButi

**Affiliations:** 1 Clinical Pharmacy, King Fahad Armed Forced Hospital, Jeddah, SAU; 2 Quality Coordination Pharmacist, King Fahad Armed Forced Hospital, Jeddah, SAU; 3 Outpatient Pharmacist, King Fahad Armed Forced Hospital, Jeddah, SAU; 4 Medication Safety Pharmacist, King Fahad Armed Forced Hospital, Jeddah, SAU; 5 Cardiac Clinical Pharmacist, King Fahad Armed Forced Hospital, Jeddah, SAU

**Keywords:** hospitalization, cardiovascular, heart failure, empagliflozin, t2dm

## Abstract

Background

Since the increasing prevalence of type 2 diabetes mellitus (T2DM), heart failure coexisting with it has had a significant impact on clinical management and prognosis. Patients with T2DM and heart failure with reduced ejection fraction (HFrEF) have increased mortality and morbidity. Empagliflozin, a sodium-glucose cotransporter-2 (SGLT2) inhibitor, is widely acknowledged to reduce cardiovascular risk in T2DM patients. We wanted to assess the composite outcomes of heart failure, cardiovascular death, and hospitalization following the start of empagliflozin therapy in the Saudi population.

Methods

This is a retrospective observational study conducted at King Fahad Armed Forces Hospital-Jeddah. We included patients aged 18 or older, male or female, with T2DM with HFrEF <40% and with a risk of cardiovascular events who were treated with empagliflozin 25 mg once daily as combination therapy and patients using other diabetic agents without empagliflozin as the comparative group.

Results

A total of 195 patients with T2DM and HFrEF who were at high risk for cardiovascular (CV) events were included in the study. Regarding gender, most of the patients (82.1%) were male with an average age of 61.28 ± 9.92. The patients were divided into 71 individuals who received empagliflozin and 124 who did not. When comparing the surgical procedure and comorbid status of the patients, coronary artery bypass graft (1.4%), coronary artery disease (5.6%), dyslipidemia (5.6%), and ischemic cardiomyopathy (0%) were found compared to the non-empagliflozin group. Meanwhile, hypertension was found to be 71.8% and ischemic heart disease was 50.7% in empagliflozin patients. Furthermore, only dyslipidemia differed significantly (p <0.001) between the empagliflozin and non-empagliflozin groups of patients. However, no significant differences were observed between the average low-density lipoprotein (p = 0.990) and high-density lipoprotein (p = 0.399). There was no significant difference observed in the primary outcome of CV deaths or hospital admission of patients between empagliflozin and non-empagliflozin. No deaths were reported in either of the comparative groups in our study.

Conclusion

In this study, there was no significant difference observed in hospital admission of the patients between the empagliflozin and non-empagliflozin groups. No cardiovascular mortality was reported in the study population. Further matched group comparative studies or placebo-controlled studies are required to compare the existing evidence of the impact of empagliflozin on T2DM patients with HFrEF and at high risk for CV deaths or hospital admission.

## Introduction

Type 2 diabetes mellitus (T2DM) is a burgeoning common metabolic disorder and a major worldwide health issue. It is an established risk factor for the development of cardiovascular diseases (CVDs) such as coronary artery disease, stroke, heart faliure (HF), arrhythmia, and hypertension (HTN) through a variety of mechanisms. T2DM and cardiovascular disease both raise the risk of death [1]. The Arab Gulf Cooperation Council countries are considered one of the regions exhibiting a high prevalence of diabetes, including Saudi Arabia, Bahrain, Qatar, Oman, Kuwait, and the United Arab Emirates, which have similar population characteristics such as lifestyle, religion, diet and income [2]. In Saudi Arabia, the prevalence rate of diabetes has been estimated to be 9%-23.7% [3,4]. HF risk was found to be higher in patients with T2DM. HF is one of the top reasons for hospital admissions globally and entails a high risk of early post-discharge mortality and rehospitalisation [5,6].

In recent years, it has been shown in research studies that inhibitors of sodium-glucose cotransporter 2 (SGLT2i) are a new generation of oral hypoglycemic agents for patients with T2DM which acts by decreasing renal glucose reabsorption, thereby boosting urinary glucose excretion [7], thus lowering plasma glucose levels and contributing to a modest hemoglobin A1C (HbA1C) level [8]. Jardiance® is in a class of medications named SGLT2s, with an approved date of August 2014 for use in patients with T2DM as an adjunct to diet and exercise to improve glycaemic control or blood glucose levels [9]. The medication, administered as either a monotherapy or an add-on therapy, is said to lower glycated hemoglobin levels in patients with T2DM, including those with stage 2 or 3 chronic kidney disease. This effect is also reportedly accompanied by weight loss and blood pressure reductions without an increase in heart rate. When combined with other anti-diabetic drugs, empagliflozin has minimal side effects [10-18]. With a reasonable risk profile, empagliflozin improves glycemic control in T2DM and lowers body weight and blood pressure in comparison to each drug alone [19]. Because the mechanism of action of empagliflozin is unrelated to the insulin pathway and beta-cell activity, there is a modest risk of hypoglycemia. However, SGLT2 inhibitors have been shown to be safe in the non-diabetic population as well. Urinary tract infection is one of empagliflozin's most frequent adverse effects [20].

Empagliflozin effect in T2DM with cardiovascular or HF on patients' mortality has not been clearly examined in Saudi population. The primary outcome of the study is to assess CVD mortality, nonfatal myocardial infarction (MI) or nonfatal stroke and assessment of heart failure as an outcome.

## Materials and methods

Patient and setting

This was a retrospective study conducted at King Fahad Armed Forces Hospital-Jeddah between 1 January 2020 and 31 December 2021 (approval REC 494). We included patients aged ≥18, male or female, T2DM with heart failure with reduced ejection fraction (HFrEF) (<40), T2DM with CVDs and risk of CV death, T2DM who were treated with empagliflozin 25 mg OD as combination therapy and patients not taking the drug in their diabetic medication. In addition, we also included patients that received at least one dose of the following treatment: diuretics, angiotensin-converting enzyme inhibitor (ACEI), hydralazine and isosorbide dinitrate for heart failure, other diabetic medication such as insulin or sulfonylurea and cholesterol-lowering agents such as the statin. Each patient’s lab results should be closely monitored for hemoglobin A1C. We documented the average level of low-density lipoprotein (LDL) and high-density lipoprotein (HDL). In addition, we recorded the ejection fraction as well as the electrocardiogram (ECG) results after each patient received the drug from the medical records. However, we excluded patients with an ejection fraction of >40%, pregnancy and those who did not attend continuous follow-up visits at the hospital.

Data collection method and study tool

We created a Microsoft Excel (Microsoft, Redmond, WA, USA) sheet of patients receiving empagliflozin between January 1, 2020, and December 31, 2021. Patients' clinical and baseline details and study outcomes such as CV death and hospital admission were extracted from the electronic medical record database. According to our data on patients receiving empagliflozin, the number of T2DM patients with cardiac events was 3,280. Based on inclusion criteria, we included 195 patients and excluded the remaining patients.

Statistical analysis

Variables were then imported into Statistical Package for Social Sciences (SPSS) version 26 (IBM Corp., Armonk, NY, USA). Means and standard deviations were used to represent the qualitative data, while numbers and percentages were used to represent the quantitative data. Descriptive statistics of baseline characteristics and comorbid disease were obtained. Normality of continuous variables such as age, LDL, and HDL levels was obtained using the Shapiro-Wilk test. The Mann-Whitney-U test was used to compare the age, LDL, and HDL between the empagliflozin and non-empagliflozin groups of patients. The Chi-square test or Fisher exact test was used to compare the CV/HF death and hospitalization in the empagliflozin and non-empagliflozin groups of patients, and the results were summarised in absolute numbers and percentages. The hospitalized patients were cross-analyzed in patients with cardiovascular comorbid diseases and between the baseline characteristics. Statistical significance was defined as a p-value of <0.05.

## Results

The baseline characteristics of the patients in the empagliflozin and non-empagliflozin groups are shown in Table 1. The study included 195 T2DM patients from King Fahad Armed Forces Hospital-Jeddah hospital, Saudi Arabia. Most of the patients were male (82.1%), and the mean age of the patients was 61.28 ± 9.92. The average age of empagliflozin patients (62.13 ± 9.88) was higher than that of non-empagliflozin patients (60.49 ± 10.03). When comparing the comorbid status of the patients, coronary artery bypass surgery (CABG) (1.4%), coronary artery disease (CAD) (5.6%), dyslipidemia (5.6%), and ischemic cardiomyopathy (ICM) (0%) were found to be in smaller proportions compared to a non-empagliflozin group of patients, while HTN was 71.8% and ischemic heart disease (IHD) was found to be 50.7% in empagliflozin patients. Only dyslipidemia differed significantly (p <0.001) between the empagliflozin and non-empagliflozin groups of patients. There was no significant difference found between the average LDL (p = 0.990) and HDL (p = 0.399) between the two comparative groups (Table 1).

**Table 1:** Comparison of baseline and clinical characteristics between the two comparative groups of patients CABG: coronary artery bypass graft; CAD: coronary artery disease; ICM: ischemic cardiomyopathy HTN: hypertension; IHD: ischemic heart disease; LDL: low-density lipoprotein; HDL: high-density lipoprotein; *: P-value < 0.05 is statistically significant

		With Empagliflozin	Without Empagliflozin	p-value
Gender	Female	10 (14.1%)	25 (20.2%)	0.336
	Male	61 (85.9%)	99 (79.8%)	
CABG	Yes	1 (1.4%)	8 (6.5%)	0.159
	No	70 (98.6%)	116 (93.5%)	
Cardiovascular disease				
CAD	Yes	4 (5.6%)	6 (4.8%)	1.000
	No	67 (94.4%)	118 (95.2%)	
HTN	Yes	51 (71.8%)	70 (56.5%)	0.046*
	No	20 (28.2%)	54 (43.5%)	
Dyslipidemia	Yes	4 (5.6%)	47 (37.9%)	<0.001*
	No	67 (94.4%)	77 (62.1%)	
IHD	Yes	36 (50.7%)	72 (58.1%)	0.370
	No	35 (49.3%)	52 (41.9%)	
ICM	Yes	0 (0%)	8 (6.5%)	0.053
	No	71 (100%)	116 (93.5%)	
Age		62.13 ± 9.88	60.49 ± 10.03	0.503
Average LDL		2.41 ± 1.14	2.41 ± 1.06	0.990
Average HDL		1.02 ± 0.27	0.99 ± 0.28	0.399

There was no significant difference observed in the hospital admission of patients between empagliflozin and non-empagliflozin (0 = 0.654). Our planned primary endpoint, such as in-hospital death, was not reported in any of the empagliflozin and non-empagliflozin groups. However, of those taking empagliflozin, over 50% of patients were not re-admitted to the hospital (Table 2).

**Table 2:** Hospitalisation status in comparison groups

		With Empagliflozin	Without Empagliflozin	p-value
Hospitalisation	Yes	31 (43.7%)	50 (40.3%)	0.654
	No	40 (56.3%)	74 (59.7%)	

The sub-analysis of patients admitted to the hospital with HF reveals that no notable or significant differences were found between patients using empagliflozin and other diabetic medications in terms of baseline, clinical and cardiovascular diseases (Table 3).

**Table 3:** Sub-analysis of patients admitted for heart failure reasons CABG: coronary artery bypass graft; CAD: coronary artery disease; ICM: ischemic cardiomyopathy HTN: hypertension; IHD: ischemic heart disease; LDL: low-density lipoprotein; HDL: high-density lipoprotein

		With Empagliflozin	Without Empagliflozin	p-value
Gender	Female	10 (14.1%)	25 (20.2%)	0.336
	Male	61 (85.9%)	99 (79.8%)	
CABG	Yes	0 (0%)	3 (6%)	0.282
	No	31 (100%)	47 (94%)	
Cardiovascular diseases				
CAD	Yes	1 (3.2%)	1 (2%)	1.000
	No	30 (96.8%)	49 (98%)	
HTN	Yes	20 (64.5%)	28 (56%)	0.448
	No	11 (35.5%)	22 (44%)	
Dyslipidemia	Yes	3 (9.7%)	0 (0%)	0.053
	No	28 (90.3%)	50 (100%)	
IHD	Yes	19 (61.3%)	35 (75%)	0.472
	No	12 (38.7%)	15 (30%)	
ICM	No	31 (100%)	50 (100%)	-----
Age		61.32 + 10.39	61.03 +10.69	0.785
Average LDL		2.38 ± 1.39	2.63 ± 1.19	0.284
Average HDL		1.02 ± 0.25	1.00 ± 0.30	0.496

## Discussion

Empagliflozin, an SGLT2 inhibitor, is widely acknowledged to reduce CV risk in T2DM patients. The benefits of empagliflozin were observed early and consistently. In our study, we evaluated the impact of empagliflozin on a composite primary outcome of CV death or hospital admission for HF in individuals with T2DM and HFrEF at high risk for CVDs and events. The prevalence of hospital admission in our included patients using empagliflozin was 43.7%. In this study, the average age of the patients was above 60, and the empagliflozin group patients were observed to have a higher average age. This may be one reason for not finding a significant difference in hospital admissions between the groups. Meanwhile, the increased risk of hospitalization for HF might be explained by the occurrence of multiple comorbidities in older adults with HFrEF [21]. In the present study, patients were reported to have multiple CV diseases like CAD, HTN, dyslipidaemia, IHD, HF, and ICM. Originally, empagliflozin was intended for the treatment of diabetes due to its glycosuric properties, yet its beneficial effects extend beyond lowering glycemia [22] and have been studied in several large clinical trials [23,24]. There is previous evidence to support the use of empagliflozin in reducing adverse events such as heart failure death, worsening heart failure events, and hospitalization in T2DM, with benefits seen early after initiation of treatment [25,26].

The SGLT2i class of antihyperglycemic medications has been shown in numerous CV outcome clinical trials to lower the risk of HF-related endpoints in patients with T2DM and either established CV disease or multiple CV risk factors. The effects of SGLT2is in patients with HFrEF, with or without T2D, have since been the subject of extensive clinical investigations [27,28]. These studies have demonstrated that dapagliflozin and empagliflozin both significantly lower hospitalization rates for HF and CV mortality. However, Lim et al. found no significant differences in clinical outcomes such as composite coronary events, composite ischemic events, hospitalization for HF, renal events, and composite HF and renal events between dapagliflozin and empagliflozin [29]. In this present study, we compared only empagliflozin as a combination therapy with a non-empagliflozin group of T2DM patients. Even if the effect of empagliflozin has been widely accepted in T2DM patients with CV risk, in a recent study conducted by Pérez-Belmonte et al. [30] regarding the resumption of empagliflozin in T2DM patients who were hospitalized for acute decompensated HF, no significant changes were found in their observational research, which included patients treated using in-hospital basal-bolus insulin vs. empagliflozin-basal insulin as their antihyperglycemic agents. No differences were observed in the major end points of the investigation, such as hospital stay duration, or in-hospital deaths. In our study, no death due to heart failure occurred in either of our groups.

Several trials have addressed the potential role of empagliflozin on lipid metabolism, and most have ended up with a negative effect. Ozcelik et al. reported that empagliflozin did not show any significant changes in lipid profiles between the pre and post-addition of the empagliflozin to the groups [31]. Despite the existing evidence, in the current study, no statistical significance was documented in LDL and HDL profiles in patients between the two comparative groups. It's interesting to note that several SGLT2 inhibitors have been shown to raise LDL cholesterol levels, but it's unclear how this relates to HDL cholesterol. Although SGLT2 medications' inhibition of glucose reabsorption may speed up compensatory lipid metabolism, subsequently lowering body weight and changing the lipid profile [32]. SGLT2 inhibitors like empagliflozin in humans lead to increased levels of LDL cholesterol and decreased levels of plasma triglycerides.

Our results need to be interpreted with some limitations. Primarily, as this research investigation is not a matched group comparative study, it is vulnerable to confounding variables. In addition, it has an inherent problem with the retrospecitve study design. A larger crossover comparison study or randomized clinical trials will be required in the future to compare with the results that are now available. Furthermore, it was an unfavourable study that produced different outcomes from prior empagliflozin research studies. Unequal sample sizes of two groups for multiple comparisons is another potential limitation of this study. Selection bias might be another limitation of the study given its retrospective nature. Lastly, the usage of different diabetic medication agents is also regarded as a limitation.

## Conclusions

In this study investigation, the ages of those who are taking empagliflozin were above average compared to the literature. Furthermore, there was no significant difference observed in the hospital admission of the patients between the empagliflozin and non-empagliflozin groups. No cardiovascular mortality was reported in the study population. No significance was found with regard to LDL and HDL profiles between the two comparative groups. Further matched group comparative studies or placebo-controlled studies are required to compare the existing evidence of the impact of empagliflozin on T2DM patients with HFrEF and at high risk for CV death.
